# Medical Therapy for Heart Failure with Preserved Ejection Fraction

**DOI:** 10.14797/mdcvj.1162

**Published:** 2022-12-06

**Authors:** Sara Varnado, Hyeon-Ju Ryoo Ali, Barry Trachtenberg

**Affiliations:** 1 Intermountain Medical Center, Murray, Utah, US; 2 Houston Methodist DeBakey Heart & Vascular Center, Houston Methodist, Houston, Texas, US; 3 J.C. Walter Methodist Transplant Center, Houston Methodist, Houston Texas, US

**Keywords:** heart failure with preserved ejection fraction, diastolic heart failure, goal directed medical therapy, sodium-glucose-cotransporter-2, pharmacotherapy

## Abstract

Heart failure with preserved ejection fraction (HFpEF) is a challenging disease state that has long been plagued by heterogeneity in diagnostic criteria and underlying etiologies. Due in part to the complexity of defining this disease and the simplistic approach of only studying medications that have shown significant improvement in heart failure with reduced ejection fraction, there have been a multitude of negative trials in this population. In the past few years, however, there have been medications that have finally shown to benefit patients with HFpEF. In particular, the blockbuster class of medications called SGLT2 inhibitors have provided a treatment option that improves outcomes in this group of patients. There is increasing focus on HFpEF research that aims to improve the phenotyping of these patients to more successfully tailor therapy and improve patient outcomes.

## Introduction

Heart failure with preserved ejection fraction is an elusive disease that has long been challenged by the lack of a unified definition and proven therapies. The original term “congestive heart failure” broadly encompassed clinical symptoms such as dyspnea and pulmonary edema but did not distinguish underlying ejection fraction (EF) and cardiac structural changes.^1^ Approximately 50% of patients with heart failure have a preserved ejection fraction.^2^ Diastolic heart failure emerged in the 1980s as a term to describe patients with normal ejection fraction but impaired left ventricular (LV) relaxation.^3^ Initially, clinicians struggled with defining and accurately diagnosing diastolic dysfunction due to the lack of standardized echocardiographic assessments. Many argued that imaging could not adequately assess for diastolic dysfunction and believed invasive hemodynamics were needed, but the latter required resources, logistics, and standardization, which were significant barriers.^3,4^

## Diagnosis

In recent years, diastolic heart failure has become known as heart failure with preserved ejection fraction (HFpEF). While the name has evolved to include a clear definition and diagnostic criteria, pathophysiologic understanding and treatment strategy has remained stagnant compared to progress in heart failure with reduced ejection fraction (HFrEF). Experts have identified the following as major gaps in the understanding of HFpEF: definition, subtypes, and end points in clinical trials.^5^ The definition has evolved from the previous definition of diastolic dysfunction in the context of an EF > 50% and is now more broadly defined as structural abnormalities resulting from high filling pressures, diastolic abnormalities, elevated biomarkers, and elevated left heart filling pressures by noninvasive and invasive hemodynamic assessment.^2,5^ This diagnosis can be made after excluding noncardiac causes of dyspnea, which can mimic heart failure.

One of the challenging aspects of the treatment of HFpEF is that unlike HFrEF, there is no clearly defined neurohormonal pathway that underlies the pathophysiology. Additionally, studies of HFpEF are challenged by pooling patients into a single “HFpEF” disease, when in reality there are varying etiologies.^5^ There are also many overlapping disease states that mimic or coexist with HFpEF, including pulmonary hypertension and infiltrative diseases such as sarcoidosis or amyloidosis. Amyloid, for example, is a disease that presents with overlapping symptoms of HFpEF but has vastly different pathophysiology, comorbidities, treatment options, and outcomes. Hahn and colleagues evaluated endomyocardial biopsies of 108 patients with HFpEF and found that 14% of their samples were positive for cardiac amyloidosis.^6^ While these patients technically meet the diagnostic criteria of HFpEF, their treatment strategy and outcomes will differ greatly. In fact, it has been speculated that the unintentional inclusion of amyloidosis patients in major HFpEF trials may have contributed to many of these trials failing to meet statistical significance.^7^

Another disease state with marked overlap is pulmonary hypertension (PH). The most common form is pulmonary hypertension due to left heart disease, which is characterized by elevated pulmonary pressures due to chronic left atrial pressure overload, leading to increased pressure in the pulmonary veins (called “post-capillary” PH). In some cases, chronic pressure overload can lead to irreversible remodeling of the pulmonary arteries and an increase in pulmonary vascular resistance, which leads to combined pre- and post-capillary PH. Invasive hemodynamics should be measured when there is a high likelihood of PH and there are implications on management and prognostication; however, there is debate regarding which variable is associated with the strongest correlation with clinical outcomes.^8,9^ The most recent 2022 guidelines are from the European Society of Cardiology, and the only class of medication with potential benefit in HFpEF-associated PH are phosphodiesterase inhibitors (PDE5i). PDE5i have been shown to improve exercise capacity, quality of life, and hemodynamics in small studies of patients with severe precapillary components to their PH and with pulmonary vascular resistance values ≥ 5 Wood units; however, there is insufficient evidence to provide a recommendation on their use.^9^ The guidelines give a class III recommendation on the use of PDE5i in post-capillary PH.

**Figure 1:**
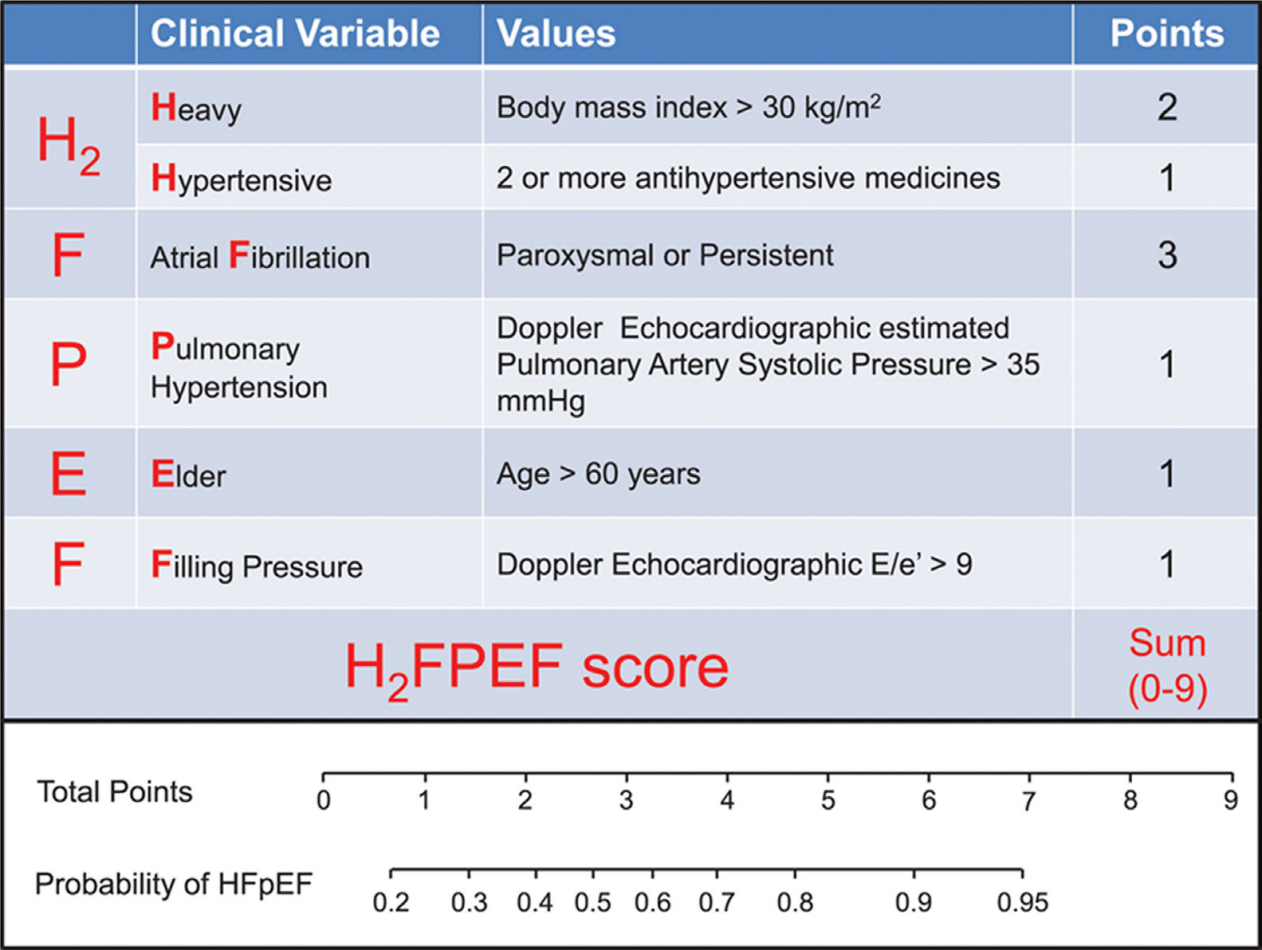
H_2_FPEF score: a simple, evidence-based approach to help in diagnosis of heart failure with preserved ejection fraction.^10^

In addition to distinguishing symptoms among various cardiac etiologies, it is important to correctly differentiate symptoms that might be attributed to HFpEF from noncardiac causes. Two recently developed scoring systems integrate variables to further differentiate HFpEF from noncardiac causes of dyspnea and can aid in determining when further diagnostic testing is needed.^10,11^ The 2022 American College of Cardiology/American Heart Association/Heart Failure Society of America Heart Failure guidelines have incorporated the HF_2_PEF score that includes six variables with a total possible score of nine points (Figure 1).^2,10^ The odds of HFpEF doubled for each 1-unit score increase (OR 1.98 [1.74–2.3]; *P* < .001) with an area under the curve of 0.841 (*P* < .001).^10^ This tool can be helpful because the overlap in diagnosis is large, but the therapeutic approach may differ significantly. It is critically important to evaluate all the components included in the score to be able to more accurately diagnose HFpEF.

## Treatment

### Renin Angiotensin Aldosterone System Inhibitors

Finding treatment strategies to improve outcomes of HFpEF has been challenging. Initial studies for HF medical therapy did not use ejection fraction (EF) as part of the inclusion criteria.^1^ The first studies that assessed all-cause mortality as the primary outcome in heart failure were the Cooperative North Scandinavian Enalapril Survival Study (CONSENSUS) and Veterans Administration Cooperative Study (V-HeFT). These studies required clinical criteria consistent with heart failure, such as dyspnea and fluid retention, but measurements of myocardial function were not required.^12,13^ A post-hoc analysis of the V-HeFT trial demonstrated that patients with a normal EF (> 45%) had a lower rate of death (8% vs 19%), leading subsequent trials to use EF of 35% to 40% as a cutoff to improve sample size and event rates. While this led to significant advancement in HFrEF research, it hindered studies for HFpEF.^1,14^ Since this time, various cutoffs (40% or 50%) have been used for HFpEF trials. There is significant variability in reported EFs read by various providers, and the cutoff values used in various studies are somewhat arbitrary, impacting diagnosis and inclusion in studies. Although EF is perhaps the most common criteria for inclusion of HFpEF trials, it is also not specific to the underlying etiology and is associated with large heterogeneity in patient phenotypes. This has likely hindered the progress in identifying beneficial therapies in specific patients.

Most clinical studies evaluating medical treatment for HFpEF have simply followed positive studies with the same drugs studied in HFrEF patient populations. Beta blockers and renin angiotensin aldosterone system (RAAS) inhibitors both reduced mortality in patients with HFrEF, and the Heart Outcomes Prevention Evaluation (HOPE) trial demonstrated that patients at high risk of cardiovascular events but without heart failure have a survival benefit from angiotensin converting enzyme (ACE) inhibitors.^15^ The use of these medications also improves outcomes in patients with diabetes, coronary artery disease, and atrial fibrillation, all of which are common comorbidities in the HFpEF population.^1,5^ Due to the broad spectrum of patients who had observed benefits from RAAS inhibition, it was hypothesized that patients with heart failure symptoms but without a reduced left ventricular ejection fraction (LVEF) would also benefit from RAAS inhibitors.

Two large trials assessed angiotensin receptor blockers (ARB) in HFpEF.^16,17^ The effects of candesartan in patients with chronic heart failure and preserved LVEF (CHARM-Preserved) trial compared candesartan to placebo, and the irbesartan in patients with heart failure and preserved ejection fraction (I-Preserve) trial compared irbesartan to placebo. The two studies both included patients with class II-IV symptoms and a recent hospitalization for heart failure, but CHARM-Preserved used an EF cutoff of ≥ 40% whereas I-Preserve only included those with an EF ≥ 45%. In CHARM-Preserved, the primary outcome (cardiovascular death or unplanned hospital readmission due to HF) was met in 22% of the candesartan patients and 24% of placebo patients, with no statistically significant difference between them (HR 0.89 [0.77–1.03]; *P* = .12). Mortality was not different between patients treated with candesartan compared with placebo (11.2% vs 11.3%), but hospitalizations for heart failure trended toward significance with candesartan (15.9% vs 18.3%, HR 0.85 [0.72–1.01]; *P* = .07), which was consistent with a prespecified adjusted analysis. In the I-PRESERVE trial, there was no statistically significant difference in the primary outcome (death from cardiovascular causes or hospital readmission for cardiovascular causes) or any secondary outcomes. The reduction in rehospitalizations seen with candesartan was not reproduced with irbesartan (HR 0.95 [0.85–1.08]; *P* = .44).

A secondary analysis was done assessing the effects on various EF ranges, including data from CHARM-Preserved, CHARM-Added (effects of candesartan in patients with chronic heart failure and reduced left-ventricular systolic function taking angiotensin-converting-enzyme inhibitors) and CHARM-Alternative (effects of candesartan in patients with chronic heart failure and reduced left-ventricular systolic function intolerant to angiotensin-converting-enzyme inhibitors) in EF ≤ 40%.^18^ Patients with an EF of 40% to 49% showed a significant reduction in the combined outcome of cardiovascular mortality and HF hospitalization (HR 0.72 [0.55–0.95]; *P* = .02) but there was no benefit seen in EF > 50%, suggesting that the patients with a moderately reduced EF may have driven the improvement in rehospitalizations. If a more conservative EF cutoff of 50% had been used, it is possible that no benefit would have been observed with candesartan. A Cochrane review done for four ARB trials in HFpEF found no significant reduction in mortality or hospitalizations (HR 0.92 [0.83–1.02]). Although the data is conflicting and modest, ARBs have been given a Class 2b recommendation in the 2022 guidelines for reductions in hospitalizations, with the caveat that benefits are largely seen in patients with EF on the lower end.^2^

Due to the benefits seen in many other populations, ACE inhibitors were also tested in HFpEF patients. The perindopril in elderly people with chronic heart failure (PEP-CHF) trial assessed perindopril in elderly people > 70 years of age with diastolic heart failure, EF > 40%, and a hospitalization within the previous 6 months.^19^ There was no difference in the primary outcome of time to death or unplanned HF hospitalization (HR 0.92 [0.70–1.21]; *P* = .545) or either of the individual components. Overall, the study had a lower event rate than anticipated and a large discontinuation rate, but ACE inhibitors do not appear to possess the same beneficial effects in HFpEF patients as a whole despite their benefits in patients with overlapping disease states.

One of the most controversial but impactful trials in HFpEF is the TOPCAT (treatment of preserved cardiac function heart failure with an aldosterone antagonist) trial.^20^ This study compared spironolactone to placebo in patients with signs and symptoms of heart failure, a LVEF ≥ 45%, and either a prior HF hospitalization or elevated natriuretic peptide level. The primary outcome was cardiovascular death, HF hospitalization, or aborted cardiac arrest. It was conducted primarily in North and South America, Russia, and Georgia. The patients included were 68 years old on average, 51% female, 63% with class II symptoms, and 71% had been hospitalized within the previous 6 months. Overall, there was no statistically significant difference in the primary outcome of death from cardiovascular causes, aborted cardiac arrest, or hospitalization for the management of heart failure with 18.6% in the spironolactone group and 20.4% in placebo (HR 0.89 [0.77–1.04]; *P* = .14). Death from cardiovascular causes (9.3% vs 10.2%) and aborted cardiac arrest (0.2% and 0.3%) were not different between the two groups, but spironolactone reduced hospitalizations (12% vs 14.2%; HR 0.83 [0.69–0.99]; *P* = .04). The surprising result from the trial was a marked regional difference in event rates.

A subgroup analysis compared the outcomes for patients in the Americas to patients in Russia and Georgia.^21^ In the Americas population, the primary outcome occurred in 29.5% of patients versus 8.9% in the Russia/Georgia patients; both rehospitalizations and mortality were significantly improved in the Americas population but not the Russia/Georgia group (*P* < .001). In the Americas, spironolactone was associated with a significant benefit (HR 0.83) but this was not the case in the Russia/Georgia arm (HR 1.10). In the overall study, there was a higher incidence of hyperkalemia and elevations in serum creatinine with spironolactone, but this was not observed in the Russia and Georgia population even though compliance was reported to be higher, thus raising concerns for actual compliance. Hence there is concern that the Russian/Georgian arm of the study was conducted poorly and that the data from this region may have negatively impacted the entire study. Due to the improvements seen in mortality and hospitalizations in the Americas, mineralocorticoid receptor antagonists are given a Class 2b recommendation in the 2022 guidelines for the reduction in hospitalizations.^2^

The angiotensin receptor and neprilysin inhibitor sacubitril-valsartan, found to have a survival benefit in patients with HFrEF compared to enalapril, was subsequently studied in HFpEF patients. In the phase 2 study, sacubitril-valsartan was shown to significantly reduce N-terminal (NT)-pro hormone brain natriuretic peptide (NT-proBNP) compared with valsartan.^22^ The outcomes trial PARAGON-HF (prospective comparison of ARNI with ARB global outcomes in HF with preserved ejection fraction) enrolled patients with EF ≥ 45% and either elevated natriuretic peptide levels or a recent hospitalization and compared sacubitril-valsartan to valsartan after a run-in period.^23^ The primary outcome of HF hospitalizations and death was not statistically significant (rate ratio 0.87 [0.75–1.01]; *P* = .06). The rates of hospitalizations were lower with sacubitril-valsartan but not statistically significant (RR 0.85 [0.72–1.00]), but these results are exploratory as the primary outcome was not met. In the prespecified subgroup analysis, patients with EF ≤ 57% significantly benefited from sacubitril-valsartan (RR 0.78 [0.64–0.95]) while those > 57% did not. Overall, sacubitril-valsartan was associated with a higher incidence of hypotension and angioedema but lower rates of hyperkalemia and increases in serum creatinine. Sacubitril-valsartan is also given a Class 2b recommendation in the 2022 guidelines to reduce hospitalizations, again emphasizing the benefits observed in patients with a lower EF.^2^

### Loop Diuretics

Elevated filling pressures are one of the cornerstone symptoms in HFpEF. Although diuretics are given to reduce congestion, there is limited data to guide their use overall and no specific studies in HFpEF. The DOSE (diuretic optimization strategies evaluation) trial is the largest diuretic trial, and while there was no EF cutoff used in the trial, the average EF of those enrolled was 35%.^24^ This trial was a 2 × 2 factorial design assessing high-dose versus low-dose diuretics and continuous versus bolus dosing of furosemide. The primary outcome was patient-perceived dyspnea using a visual analog scale. There was no difference in the primary outcome with bolus versus continuous infusion (*P* = .47) but high dose was associated with a nonsignificant improvement in symptoms (*P* = .06) compared with low dose. There were no differences in secondary outcomes with bolus versus continuous infusion, but high dose was associated with an improvement in net fluid loss at 72 hours (*P* = .001) and change in weight at 72 hours (*P* = .01). From this trial, we learned that patients admitted with volume overload benefit from at least 2.5-times their home-dose equivalent in intravenous diuretics. Alternative strategies for elevated filling pressures include implantable systems for chronic monitoring of intracardiac and pulmonary artery pressures such as CardioMEMS™ heart sensor, which looked at outcomes in New York Heart Association (NYHA) class III heart failure patients in the CHAMPION trial.^25^ While this is beyond the scope of this paper, this strategy is associated with a significant reduction in HF hospitalizations in patients with HFpEF and HFrEF and also has shown benefits recently in NYHA class II patients.^26^

### Sodium-Glucose Cotransporter-2 Inhibitors

Trailing the multiple trials for pharmacotherapies that have failed to demonstrate effectiveness in improving outcomes in HFpEF, sodium-glucose cotransporter-2 (SGLT2) inhibitors have forever changed this paradigm. After the EMPEROR-REDUCED trial (empagliflozin outcome trial in patients with chronic heart failure and a reduced ejection fraction) demonstrated the incremental improvement in outcomes for HFrEF patients when added to guideline-directed medical therapy, the EMPEROR-PRESERVED trial (empagliflozin outcome trial in patients with chronic heart failure with preserved ejection fraction) proved something similar for HFpEF patients. In this randomized double-blind placebo-controlled trial, patients with EF > 40% with a clinical diagnosis of chronic heart failure based on elevated NT-proBNP (≥ 900 pg/mL or ≥ 300 pg/mL for patients with or without atrial fibrillation, respectively) were enrolled and randomized to receive either 10 mg empagliflozin or placebo.^27^ This trial succeeded in demonstrating a statistically significant 21% reduction in primary composite outcomes including heart failure outcomes or cardiovascular death (*P* < .001). In fact, this trial demonstrated renal-protective benefits with significantly lower progression of chronic kidney disease (average change in estimated glomerular filtration rate per year). While this trial failed to demonstrate improvement in functional status, other trials since then have demonstrated improvement in functional status.^28^

**Figure 2:**
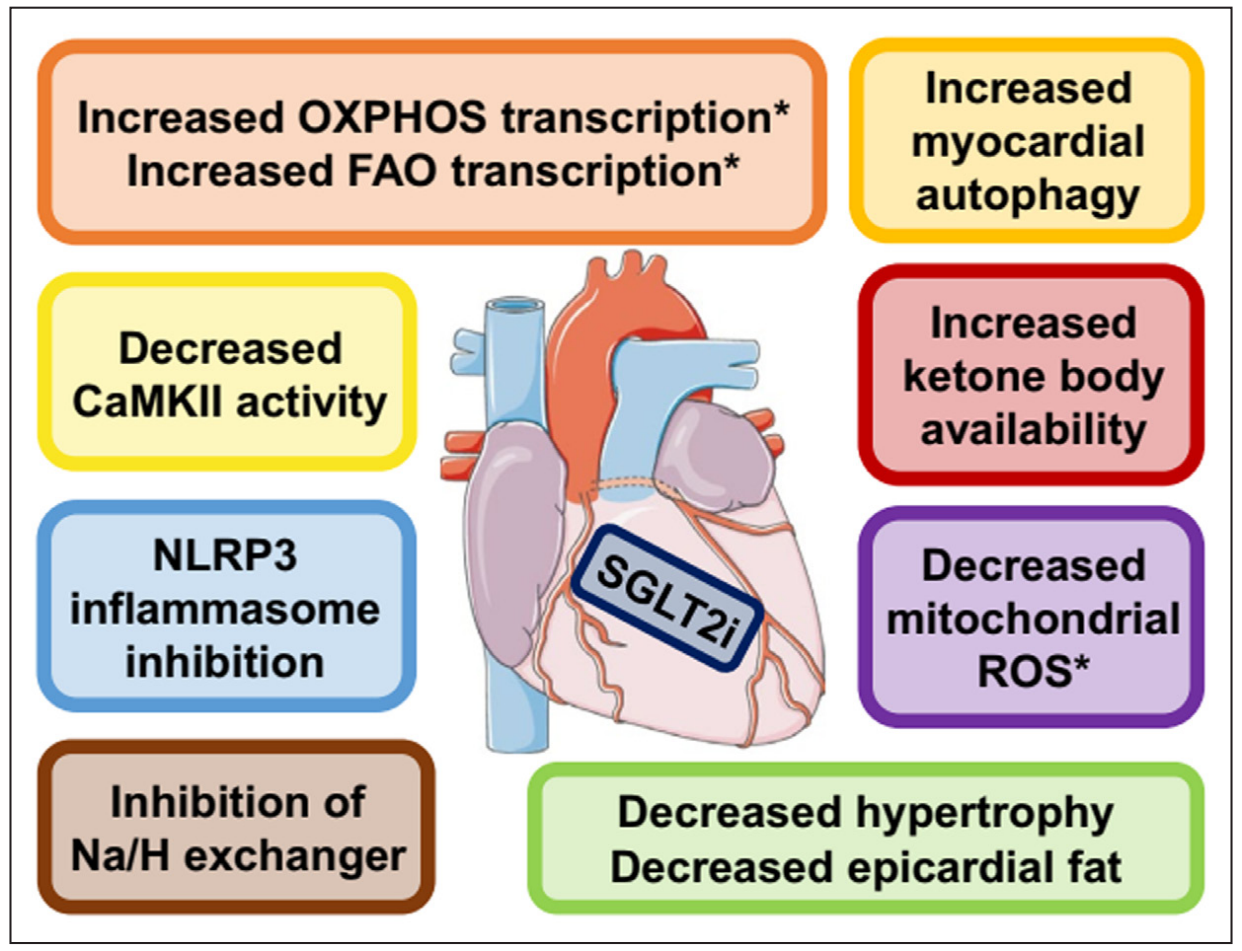
Effects of SGLT2 inhibition at the myocardial level.^30^ CaMKII: calcium/calmodulin-dependent protein kinase II; FAO: fatty acid oxidation; Na/H: sodium/hydrogen; NLRP3: NLR family pyrin domain-containing 3; OXPHOS: oxidative phosphorylation; ROS: reactive oxygen species; and SGLT2i: sodium-glucose cotransporter-2 inhibition * Signifies myocardial processes.

**Figure 3:**
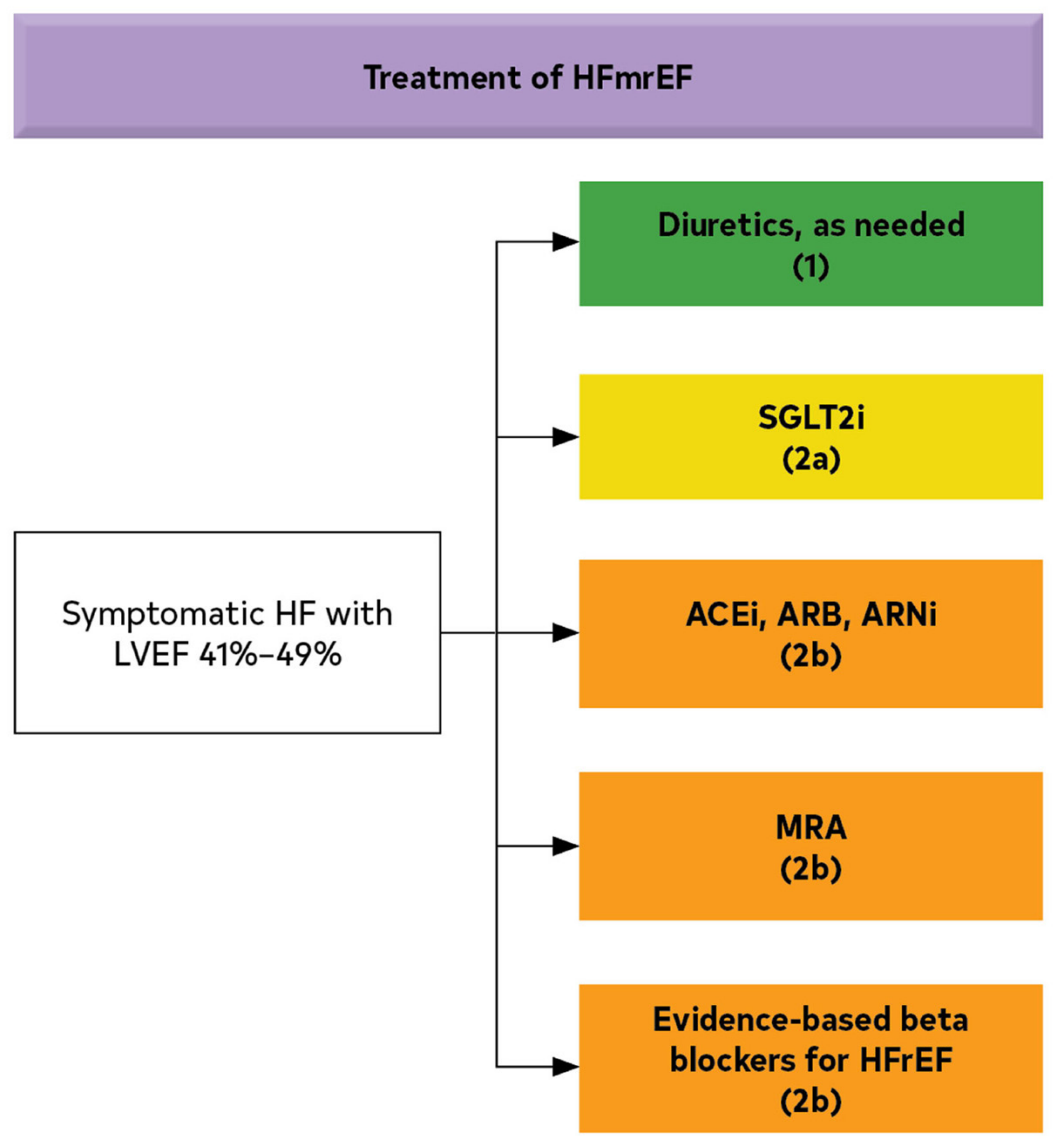
American College of Cardiology/American Heart Association 2022 Guidelines for the Management of Heart Failure.^2^ HFpEF: heart failure with preserved ejection fraction; LVEF: left ventricular ejection fraction; SGLT2: sodium-glucose cotransporter-2; ARNI: angiotensin receptor/neprilysin inhibitor; MRA: magnetic resonance imaging; ARB: angiotensin receptor blocker * greater benefit in patients with LVEF closer to 50%.

More recently, the DELIVER-HF trial (dapagliflozin evaluation to improve the lives of patients with preserved ejection fraction heart failure) has demonstrated treatment with dapagliflozin 10 mg daily in primary outcomes (worsening HF event or cardiovascular death) in patients with EF > 40% and with and without recent HF hospitalization.^29^ This latter trial, presented at the latest European Society of Cardiology meeting in 2022, further confirmed the survival benefits in patients treated with SGLT2 inhibitors. Proposed mechanisms of action for SGL2 inhibitors are, in fact, not related to the RAAS inhibition as is traditionally thought in HFrEF pharmacotherapies. While many have hypothesized that the glucosuric effects may augment diuresis, thus improving decongestion, animal models have shown direct impact of SGLT2 inhibition in cardiometabolics and cardiac energetics (see Figure 2).^30^ Figure 3 summarizes the 2022 guideline recommendations on treatment of HFpEF.^2^

**Figure 4:**
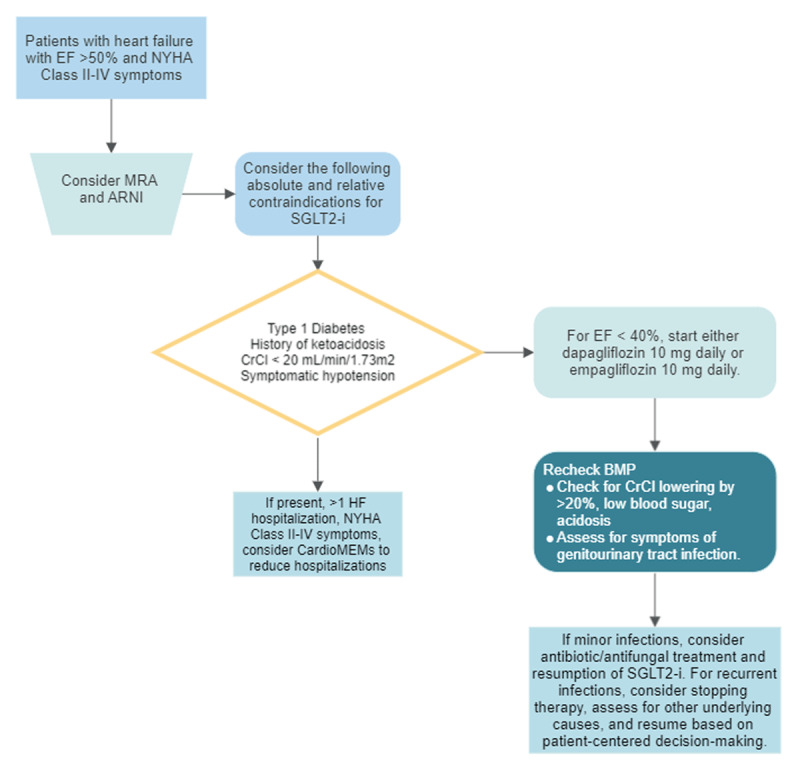
Initiation and up-titration of empagliflozin in patients with heart failure with preserved ejection fraction. EF: ejection fraction; NYHA: New York Heart Association; SGLT2-i: sodium-glucose transporter-2 inhibitor; CrCl: creatinine clearance; BMP: basic metabolic panel.

Figure 4 outlines some practical tips in initiating and monitoring the effects of SGLT2 inhibitors. Absolute contraindications for initiation of SGLT2 inhibitors include patients with type 1 diabetes and history of diabetic ketoacidosis given their elevated risks of developing ketoacidosis. Relative contraindications include hypotension (up to 6.6% in EMPEROR-PRESERVED) and acute renal failure (up to 12.1% in EMPEROR-PRESERVED). Despite concerns regarding worsening renal function in patients with chronic kidney disease (CKD), a recent study demonstrated delay in progression of CKD in this population.^31^ Patients with CKD may experience up to a 10% to 15% decline in creatinine clearance; however, more than a 20% decline should raise concerns for acute renal failure, and SGLT-2 inhibitors should be stopped.^32^ Lastly, SGLT2 inhibitors may increase the risk of genitourinary infections. If this occurs, the SGLT2 inhibitor should be stopped and the infection treated with an antibiotic or antifungal. Clinicians should consider reinitiating the SGLT2 inhibitor after the infection resolves based on patient-centered decision-making.

## Conclusion

While HFpEF patients have not had the robust armamentarium that HFrEF patients have, evidence-based options are finally available to support the management of these complex patients. The newest blockbuster class of SGLT2 inhibitors are proving to have dramatic effects across a wide array of cardiovascular patients, and for the first time we are seeing signs of a pharmacotherapeutic benefit in HFpEF patients. The use of the newly developed scoring systems will be valuable in not only diagnosing HFpEF but also improving the homogeneity of populations enrolled in our clinical trials. Using EF cutoffs with evidence of diastolic dysfunction is a nonspecific inclusion criterion and could have led to missed opportunities in accurately testing the effects of medications, such as spironolactone and sacubitril-valsartan. As we continue to improve the phenotyping of HF and our understanding of the diversity of phenotypes within HFpEF, the future holds promise for more individualized and targeted therapies based on the true molecular pathophysiology of diastolic dysfunction in select populations.

## Key points

- The HF_2_PEF score is a validated tool that can be used to evaluate cardiac from noncardiac causes of dyspnea and aid in the diagnosis of heart failure with preserved ejection fraction (HFpEF).
- Angiotensin receptor blockers, aldosterone antagonists, and angiotensin receptor-neprilysin inhibitors (sacubitril/valsartan) can be considered in HFpEF, especially in patients with ejection fractions closer to 50%.
- The newest class of medications, sodium-glucose cotransporter-2 inhibitors, are the first to show improved outcomes in HFpEF, with a reduction in heart failure hospitalizations and mortality, and should be utilized in all HFpEF patients unless a contraindication exists.
- Further effort to improve diagnosis and identify specific phenotypes of HFpEF are needed to tailor trials and therapy since significant heterogeneity exists across the HFpEF spectrum.
